# Effects of Sugammadex on the Coagulation Profile of Living Liver Donors Undergoing Hepatectomy: A Case-Control Study

**DOI:** 10.3390/medicina61030378

**Published:** 2025-02-22

**Authors:** Duygu Demiroz, Yusuf Ziya Colak, Sami Akbulut, Oya Olcay Ozdes, Muharrem Ucar, Mehmet Ali Erdogan, Serdar Karakas, Nurcin Gulhas

**Affiliations:** 1 Department of Anesthesiology and Reanimation, Faculty of Medicine, Inonu University, 44280 Malatya, Turkey; 2 Department of Surgery, Liver Transplant Institute, Faculty of Medicine, Inonu University, 44280 Istanbul, Turkey

**Keywords:** living donor liver transplantation, living donor hepatectomy, coagulation parameters, sugammadex, neostigmine

## Abstract

*Background*: The most important concern regarding living donor liver transplantation is the safety of living liver donors, of which anesthesia management is an important part. Sugammadex, which has recently been used frequently for the reversal of neuromuscular blockade, is known to cause adverse effects on the coagulation profile. This study seeks to assess the impact of neostigmine and sugammadex on coagulation parameters in living liver donors following hepatectomy. *Methods:* We compared the demographic, clinical, and coagulation parameters of 209 living liver donors who received sugammadex (2 mg/kg) for neuromuscular blockade reversal during donor hepatectomy procedures from January 2018 to July 2022, with 209 patients who received neostigmine (50 g/kg) for the same purpose during the same timeframe. We compared the following parameters: age, gender, prothrombin time (PT), partial thromboplastin time (PTT), international normalized ratio (INR), hemoglobin (Hb), platelet count, ICU stay, hospital stay, and relaparotomy for bleeding and other causes. *Results:* Demographic data and preoperative biochemical values were similar in both groups. PT (*p* = 0.004) and aPTT (*p* < 0.001) values were significantly longer in the postoperative period in both groups; the difference between preoperative and postoperative PT (*p* = 0.009) and aPTT (*p* < 0.001) was significantly higher in the sugammadex group. However, neither group showed any elongation beyond the reference range. The sugammadex group had an elevated postoperative platelet count (*p* = 0.040). The duration of patients’ stay in the ICU was significantly shorter in the sugammadex group (*p* < 0.001). Conclusion: The prolonged aPTT and PT associated with sugammadex did not lead to any postoperative bleeding complications. The sugammadex group significantly reduced the duration of ICU stays, while the hospital stays remained comparable. Further multicentric prospective randomized studies should support our study’s findings, which demonstrate the safe use of low-dose sugammadex.

## 1. Introduction

Since Strazl et al. [1] carried out the first successful liver transplantation (LT) in 1967, LT has emerged as the preferred treatment for numerous liver diseases. While most LTs performed in Western countries are deceased donor liver transplantations, most LTs performed in Middle Eastern and Asian countries are living donor liver transplantations [2]. In Western countries, the primary concern regarding living donor liver transplantation is donor safety, as numerous precautions are necessary to prevent any complications during living donor hepatectomy in healthy living liver donors. Despite all precautions, the literature has reported significant living liver donor complications and even deaths. We can group the complications seen in living liver donors under two main headings: surgical and medical. One of the most important medical complications is harvesting a large portion of the total liver volume and temporary coagulation abnormalities caused by drugs used in perioperative anesthesia management [3,4,5]. Previous studies have indicated that some drugs used in the perioperative anesthetic management of patients undergoing major liver surgery may induce the aforementioned postoperative coagulation problems [6].

Maintaining neuromuscular blockade is an important part of general anesthesia to provide endotracheal intubation or to facilitate surgery [7]. Reversal of neuromuscular blockade is usually achieved using neostigmine and sugammadex. Sugammadex, a synthetic gamma-cyclodextrin, is an alternative pharmacological drug that can reverse neuromuscular blockades without the adverse effects caused by anticholinesterase drugs. Since its introduction, sugammadex has demonstrated significant efficacy and safety, ushering in a new era of patient safety in anesthesiology [8,9,10]. While vomiting, tachycardia, hypotension, and anaphylactic reactions are the most commonly reported adverse effects of sugammadex, some studies have demonstrated its ability to prolong prothrombin time (PT) and activated partial thromboplastin time (aPTT) [11,12,13]. Neostigmine, which reversibly inhibits the enzyme acetylcholinesterase, has been routinely used as an agent to reverse neuromuscular blockade. Neostigmine causes undesirable muscarinic side effects such as bronchospasm, intestinal hypermotility, and bradycardia. There are many studies, including meta-analyses, showing that neostigmine does not cause a change in the coagulation profile and even causes less bleeding than other agents [14].

We conducted a comprehensive search of the current medical literature and found no studies comparing the effects of sugammadex and neostigmine on postoperative coagulation components in living liver donors undergoing living donor hepatectomy. In our study, we aimed to compare the effects of sugammadex and neostigmine on coagulation profiles and early postoperative complications in this group of patients.

## 2. Materials and Methods

### 2.1. Study Period and Place

The demographic, clinical, biochemical, and early postoperative features of 1089 living liver donors aged ≥18 years who underwent living donor hepatectomy for living donor liver transplantation under elective conditions between January 2018 and July 2022 at the Inonu University Liver Transplantation Institute were retrospectively analyzed.

### 2.2. Definition of Groups and Exclusion Criteria

According to the study’s purpose, we designated 209 of the 230 living liver donors who used sugammadex for neuromuscular blockade reversal during the aforementioned study period as the “case group”. We determined the “control group” of this study to be 209 of the 859 living liver donors who used neostigmine for the reversal of neuromuscular blockade in the same period. We selected the control group using a 1:1 matching method, based on age and gender variables, and a random numerator. Living liver donors with coagulation problems or those on anticoagulant therapy were excluded. Moreover, donors lacking enough medical records, those requiring intraoperative blood transfusions—including two patients who developed right hepatic vein injury during dissection and required blood transfusion—and those requiring further doses of sugammadex or neostigmine were also excluded from the study.

### 2.3. Definition of Study

Living liver donors were grouped into two categories according to the drug used to reverse rocuronium-induced neuromuscular blockade. Living liver donors who received 2 mg/kg sugammadex were designated the “sugammadex group,” while those who received atropine 10 µg/kg and neostigmine 50 µg/kg were designated the “neostigmine group”.

### 2.4. Data Definitions

We recorded the patient’s age, gender, and preoperative and postoperative biochemical values. We routinely took postoperative investigations immediately after the surgery procedure finishes (in 1 h), which include prothrombin time (PT), partial thromboplastin time (PTT), international normalized ratio (INR), hemoglobin (Hb), and platelet count [15]. We named the preoperative values as t0 and the postoperative values as t1. The difference between t0 and t1 was calculated and recorded as the t-difference. Reference intervals for biochemical parameters in our institution are as follows: PT: 10–14 s, aPTT: 26–35 s, INR: 0.8–1.2, hemoglobin male/female: 14–17.4/12–16 g/dL, platelet: 250–400 × 10 3/µL. We used these reference ranges in the analysis of patient outcomes. Additionally, we recorded and evaluated the number of living liver donors who underwent relaparotomy in the postoperative period due to bleeding and other reasons, as well as the length of their stay in the intensive care unit (ICU) and hospital.

### 2.5. Perioperative Anesthetic Management

We routinely monitored all patients with a 3-lead electrocardiogram, pulse oximetry, and invasive arterial pressure. Additionally, we monitored the depth of anesthesia and neuromuscular blockade using the bispectral index (BIS) and train-of-four (TOF), respectively. We routinely induced general anesthesia in all patients using intravenous fentanyl at 1–2 g/kg, 4–5 mg/kg thiopental sodium, and 0.6 mg/kg rocuronium bromide. We maintained anesthesia using sevoflurane and administered 0.1 mg/kg/hour rocuronium bromide. The clinician administered 0.08 mg/kg morphine sulfate for postoperative analgesia. At the end of the operation, patients were extubated with 2 mg/kg sugammadex (Bridion^®^; MSD, Haarlem, The Netherlands) or neostigmine 50 µg/kg and atropine 10 µg/kg, at the clinician’s discretion. During the postoperative period, the intensive care unit observed all patients and provided them with patient-controlled analgesia (morphine).

### 2.6. Surgical Management

The same team at our Liver Transplantation Institute applied a standardized living donor hepatectomy protocol. We performed the laparotomy using a reverse “L” incision, exploring the abdomen and taking protocol biopsies from both liver lobes. After dissecting the gallbladder, we performed cholangiography by administering contrast material from the cystic duct to visualize the anatomy of the biliary tract. Next, we freed the relevant lobe from the surrounding tissues based on whether we would apply a right, left, or left lobe lateral segment resection. The Cavitron Ultrasonic Surgical Aspirator (CUSA) (CUSA Excel, Valleylab Inc., Boulder, CO, USA) completed the hepatectomy with parenchymal transection. Once we closed the stumps, we evaluated the remnant biliary tracts with cholangiography and achieved hemostasis.

### 2.7. Study Protocol and Ethics Committee Approval

This descriptive and analytic study involving human participants was in accordance with the ethical standards of the institutional and national research committee and with the 1964 Helsinki Declaration, its later amendments, or comparable ethical standards. The Inonu University Institutional Review Board (IRB) granted ethical approval for the non-interventional studies, with a date of 2022 and no. 3746. Each participant gave verbal and written consent before the LT procedure. We utilized the STROBE (Strengthening the Reporting of Observational Studies in Epidemiology) guideline to assess the likelihood of bias and the overall quality of this study [16].

### 2.8. Statistical Analysis

We used IBM SPSS Statistics for Windows, version 25.0 (IBM Corporation, Armonk, NY, USA), for statistical analysis. We tested the distribution of data using the Shapiro–Wilk test. We express categorical variables as numbers and percentages, and continuous variables as means, standard deviations, or medians with the interquartile range, as appropriate. We performed comparisons between the groups using the chi-squared test or Fisher exact test for categorical variables, and the Student t-test or Mann–Whitney U test for continuous variables, as appropriate.

## 3. Results

Table 1 shows the preoperative demographic parameters and coagulation profiles of 408 patients included in the study. Accordingly, no statistically significant difference was found between the sugammadex and neostigmine groups in terms of age (*p* = 0.110), gender (*p* = 0.763), PT (*p* = 0.508), aPTT (*p* = 0.818), INR (*p* = 0.430), hemoglobin (*p* = 0.901), and platelet (*p* = 0.898) variables.

Table 2 compares the coagulation profiles of the sugammadex and neostigmine groups before surgery (t0), right after surgery (t1), and the difference between the two measurements (t-difference). The sugammadex group and the neostigmine group showed prolonged PT times in the biochemical parameters evaluated in the first hour (t1) after completion of living donor hepatectomy, with the prolongation in the sugammadex group being statistically significant (*p* = 0.004). When the difference between PT t0 and PT t1 was evaluated, it was observed that there was a statistically significant difference in the sugammadex group from the neostigmine group (*p* = 0.009).

Examining the biochemical values at the first hour (t1) after living donor hepatectomy, both groups showed a prolonged aPTT value compared to the preoperative values. However, this prolongation was more pronounced in the sugammadex group (*p* < 0.001). Subsequent to the examination of the difference between the t0 and t1 values, it was determined that there was a statistically significant prolongation in the sugammadex group (*p* < 0.001). However, this prolongation was within the reference values.

Preoperative and postoperative INR values were similar in both groups. The evaluation of the preoperative and postoperative INR difference (t-difference) revealed a significantly higher difference in the neostigmine group (*p* = 0.033). The results in both groups were within the normal reference range.

Preoperative and postoperative hemoglobin values were similar in both groups. Evaluation of the difference between t0 and t1 revealed a similar decrease in hemoglobin in both groups (*p* = 0.781). Examining the platelet values of the groups revealed similar t0 values. The sugammadex group experienced an increase in platelet values after the living donor hepatectomy living donor hepatectomy, whereas the neostigmine group experienced a decrease (*p* = 0.040). We found the difference between platelet values t0 and t1 to be statistically significant (*p* = 0.015). Both groups’ hemoglobin and platelet values fell within the reference range.

Table 3 shows the comparison of both groups in terms of some early postoperative clinical features. Evaluation of the living liver donors’ postoperative period revealed that the sugammadex group did not perform relaparotomy due to bleeding. One living liver donor in the neostigmine group underwent another operation due to bleeding. Twelve living liver donors in the sugammadex group and fifteen in the neostigmine group underwent relaparotomy for non-bleeding reasons. There was no statistically significant difference (*p* = 0.108). When we evaluated the duration of the postoperative ICU stay for the living liver donors, it was 2.4 ± 0.9 days in the sugammadex group, and it was found to be significantly lower than in the neostigmine group (*p* = 0.000). We evaluated the postoperative hospital stay and found similar results in both groups (*p* = 0.882).

**Postoperative Outcomes and Complications**

No living liver donors in the current study encountered significant postoperative problems, including reintubation, aspiration pneumonia, or pulmonary embolism, within the 30-day postoperative follow-up period. Moreover, no donors experienced serious bleeding complications, except for a single case in the neostigmine group. Additionally, there were no instances of severe morbidity or mortality observed throughout the follow-up period, highlighting the overall safety and favorable short-term outcomes in this study population.

## 4. Discussion

In this study, we evaluated the effects of sugammadex versus neostigmine on coagulation factors in living liver donors who have undergone living donor hepatectomy procedures. We observed a significant prolongation of PT and aPTT in living liver donors using sugammadex compared to neostigmine. However, we concluded that this prolongation does not cause clinical complications related to bleeding. As a secondary outcome, we observed that patients who used sugammadex had shorter ICU stays.

Sugammadex has well-established efficacy and safety. The European Union approved sugammadex for use in 2008, and the United States approved it in 2015. Sugammadex acts by removing the concentration gradient of rocuronium from the neuromuscular junction [17]. Sugammadex is associated with lower side effects compared to neostigmine [14,18]. When sugammadex was first introduced, it was stated in preclinical in vivo and in vitro studies that it could cause 10% to 20% prolongation in PT and aPTT [19]. Studies conducted with healthy subjects have shown prolongation of aPTT and PTT in patients who received 4 and 16 mg/kg sugammadex [13,20].

The liver is the organ primarily responsible for the synthesis of factors involved in the coagulation and fibrinolytic pathways as well as the regulation of activators and inhibitors. Therefore, the liver’s functional capacity closely correlates with the functionality of the coagulation system. Depending on the extent of liver resection, the amount of bleeding during surgery, and the functional capacity of the remaining liver tissue, the risk of coagulopathy may increase [21,22]. Based on the data in the literature, we assumed in the present study that the use of sugammadex would cause a more pronounced prolongation of coagulation values in patients undergoing major liver resection. Despite lacking a clinical reflection, the study’s results biochemically confirmed our hypothesis. Clarifying even a theoretical bleeding risk is crucial, as safety should always be the top priority in living liver donors. In our study, we excluded living liver donors with intraoperative bleeding requiring blood transfusion because transplantation directly alters clotting factors, which would bias the study results.

Carron et al. [18] conducted a meta-analysis of 13 articles and found that sugammadex was better than neostigmine because it reversed neuromuscular blockade more quickly and reliably and had a lower risk of side effects. When looking at the details of this study, sugammadex was associated with lower rates of global side effects, respiratory side effects, cardiovascular side effects, and postoperative weakness. However, the agents were similar in terms of postoperative nausea and vomiting, pain, neurologic side effects, and changes in laboratory tests. The results of the study presented here are similar to those of Carron et al. [18] in many respects, but the neostigmine group appears to be superior in terms of blood parameters. However, we believe that further prospective randomized studies in living liver donors are necessary, given the characteristics of the patient group. Samara et al. [23] prepared a systematic review of nine studies, and the authors stated that sugammadex caused a transient increase in PT and aPTT values, but this increase was not clinically significant, which is in direct agreement with the study presented here.

Sugammadex can reverse neuromuscular blockade maintained by continuous infusion of rocuronium in LT recipients. Liang et al. [24] conducted a comparison between the use of sugammadex and neostigmine in 241 patients undergoing LT. The authors reported that sugammadex caused a prolongation of aPTT, but they found no difference between the groups in terms of postoperative bleeding, reoperation, and hospital stay. Deana et al. [6] examined the use of sugammadex and neostigmine in 41 liver transplant patients. They found that sugammadex reversed neuromuscular blockade faster and did not cause significant changes in coagulation parameters. Monse et al. [10] have shown that sugammadex can improve perioperative efficiency by decreasing operating room length and postoperative recovery time compared to neostigmine. Fiorda Diaz et al. [9] indicated that sugammadex not only influences coagulation parameters but is also linked to rapid neuromuscular blockade reversal, which may diminish postoperative complications and enhance perioperative recovery. Moon et al. [25] compared pyridostigmine and sugammadex in living liver donors who underwent living donor hepatectomy, and they showed that there was no significant difference between the groups in terms of a drop in hemoglobin level, a drop in platelet count, and a prolongation in PT and aPTT values. The authors also stated that sugammadex did not increase bleeding tendency in living liver donors with a high risk of postoperative coagulopathy; it reduced the duration of anesthesia and hospital stay, indicating its safe use in living liver donors. The patient population and study results of Moon et al. [25] closely resemble the study we present. However, our study revealed a greater prolongation in PT, aPTT, and INR in the sugammadex group, even though the clinical results did not reflect this.

Another retrospective study found that sugammadex at doses of 2 and 4 mg/kg did not affect clotting times and was safe to use in surgical patients at high risk for postoperative bleeding [26]. In contrast, Chang et al. [27] observed an increase in clotting time in surgical patients using sugammadex 4 mg/kg and stressed the need for caution when using it in high-risk postoperative bleeding cases. There are inconsistent results due to the differences in patient groups and sugammadex doses in the studies. We administered 2 mg/kg sugammadex in our clinic for donor safety. In our study, we observed prolongation in the postoperative coagulation parameters in both groups based on the baseline values. We observed a significant prolongation of PT and aPTT at a dose of 2 mg/kg sugammadex compared to neostigmine. However, this elongation was within the reference range. This prolongation did not lead to complications related to hemoglobin and postoperative bleeding. We believe that these results are not solely due to sugammadex, but also to the temporary impairment in the synthesis of coagulation factors caused by the major hepatectomy.

Early extubation, brief ICU duration, and prompt mobilization are factors that influence postoperative morbidity and death. Two studies indicated that sugammadex can diminish postoperative complications, expedite extubation durations, and lower the duration of stay in the postanesthetic care unit [28,29]. The present study also indicated that sugammadex considerably decreased ICU stay in comparison to neostigmine. This result supports past research indicating that sugammadex promotes expedited recovery of neuromuscular blockade, resulting in prompt extubation and enhanced postoperative stability. Enhanced recovery from neuromuscular blockade may diminish the likelihood of postoperative respiratory problems, hence reducing the necessity for extended ICU stay. Studies examining the effects of sugammadex on perioperative efficiency have yielded comparable results [9,10]. Additional prospective trials are necessary to investigate the long-term effects of sugammadex on ICU and hospital durations.

## 5. Limitations

The current study has some limitations. As a retrospective analysis, it does not allow for the assessment of the long-term effects of coagulation or the risk of late-phase bleeding. Furthermore, we were unable to assess recovery times and complications not documented in the electronic medical record. Our results suggest that sugammadex increases aPTT and PT without increasing postoperative bleeding. However, these results need to be confirmed by prospective, multicenter studies in the future to find out what the long-term effects are. To reduce selection bias, we used a 1:1 random match method for the control group based on age and gender. However, a well-designed prospective study would provide more definitive evidence regarding the effect of sugammadex on coagulation parameters and postoperative outcomes in living liver donors.

## 6. Conclusions

Although sugammadex has been shown to prolong coagulation parameters, it has not been shown to cause bleeding in the early postoperative period or prolong hospitalization; unexpectedly, it has been correlated with a decrease in ICU stay. Further multicentric prospective randomized studies should support this study, which demonstrates the safe use of low-dose sugammadex.

## Figures and Tables

**Table 1:** Patient characteristics and preoperative laboratory data.

Variables	Sugammadex Group (*n* = 209)	Neostigmine Group (*n* = 209)	*p*
Age (years)	30.8 ± 7.8	32.1 ± 8.1	0.110
Gender (Female) (%)	79 (37.8)	82 (39.2)	0.763
PT (s)	11.9 ± 0.7	11.8 ± 1.0	0.508
aPTT (s)	26.1 ± 3.2	26.0 ± 3.7	0.818
INR	0.9 ± 0.06	0.9 ± 0.07	0.430
Hemoglobin (g/dL)	14.5 ± 1.9	14.5 ± 2.0	0.901
Platelet (×10 ^3^/µL)	271.1 ± 57.1	270.4 ± 65.7	0.898

**Table 2:** Comparison of sugammadex and neostigmine groups in terms of coagulation parameters.

Variables	Sugammadex Group (*n* = 209)	Neostigmine Group (*n* = 209)	*p*
PT t0	11.9 ± 0.7	11.8 ± 1.0	0.508
PT t1	14.7 ± 1.9	14.1 ± 2.5	0.004
PT (t-difference)	+2.7 ± 1.9	+2.2 ± 2.5	0.009
aPTT t0	26.1 ± 3.2	26.0 ± 3.7	0.818
aPTT t1	30.3 ± 3.6	26.7 ± 3.4	<0.001
aPTT (t-difference)	+4.2 ± −4.6	+0.7 ± 3.5	<0.001
INR t0	0.9 ± 0.06	0.9 ± 0.07	0.430
INR t1	1.15 ± 0.1	1.19 ± 0.2	0.064
INR (t-difference)	+0.1 ± 0.1	+0.2 ± 0.2	0.033
Hemoglobin t0	14.5 ± 1.9	14.5 ± 2.0	0.901
Hemoglobin t1	13.8 ± 2.2	13.7 ± 2.5	0.752
Hemoglobin (t-difference)	0.7 ± 1.0	0.7 ± 2.3	0.781
Platelet t0	271.1 ± 57.1	270.4 ± 65.7	0.898
Platelet t1	278.5 ± 73	263.5 ± 7.6	0.040
Platelet (t-difference)	+7.4 ± 57.1	−6.8 ± 62.1	0.015

**Table 3:** Comparison of sugammadex and neostigmine groups in terms of postoperative variables.

Variables	Sugammadex Group (*n* = 209)	Neostigmine Group (*n* = 209)	*p*
ICU stay (days)	2.4 ± 0.9	2.9 ± 1.7	<0.001
Hospital stay (days)	15.5 ± 8.5	15.4 ± 8.5	0.882
Relaparotomy for bleeding (*n*)	0	1	0.999 *
Relaparotomy for others (*n*)	12	15	0.691 *

*: Fisher’s exact test.

## Data Availability

The datasets analyzed during the current study are available from the corresponding author upon reasonable request.

## Footnotes:

Publisher’s Note: MDPI stays neutral with regard to jurisdictional claims in published maps and institutional affiliations.
